# μ_2_-Acetone-diacetone[μ_3_-tris­(trifluoro­meth­yl)methano­lato]bis­[μ_2_-tris­(trifluoro­meth­yl)methano­lato]trilithium

**DOI:** 10.1107/S1600536810044685

**Published:** 2010-11-06

**Authors:** Hannes Vitze, Hans-Wolfram Lerner, Michael Bolte

**Affiliations:** a Institut für Anorganische Chemie, J. W. Goethe-Universität Frankfurt, Max-von-Laue-Strasse 7, 60438 Frankfurt/Main, Germany

## Abstract

The title compound, [Li_3_(C_4_F_9_O)_3_(C_3_H_6_O)_3_], features an open Li/O cube with an Li ion missing at one corner. Three of the four bridging O atoms of the cube carry a fluorinated *tert*-butyl residue, whereas the fourth is part of an acetone mol­ecule. Two of the Li atoms are further bonded to a non-bridging acetone mol­ecule. Two of the lithium ion coordination geometries are very distorted LiO_4_ tetra­hedra; the third could be described as a very distorted LiO_3_ T-shape with two distant F-atom neighbours. The Li⋯Li contact distances for the three-coordinate Li^+^ ion [2.608 (14) and 2.631 (12) Å] are much shorter that the contact distance [2.940 (13) Å] between the tetra­hedrally coordinated species.

## Related literature

For background to weakly coordinating ligands, see: Kern *et al.* (2008 ▶); Reisinger *et al.* (2007 ▶); Lerner *et al.* (2002 ▶, 2005 ▶). For a comparable cage structure with an Mg/O skeleton, see: Zechmann *et al.* (2001 ▶).
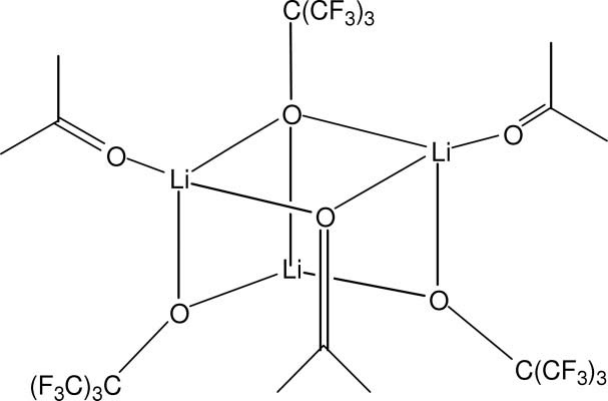


## Experimental

### 

#### Crystal data

[Li_3_(C_4_F_9_O)_3_(C_3_H_6_O)_3_]
*M* *_r_* = 900.17
Monoclinic, 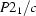

*a* = 20.6939 (17) Å
*b* = 11.594 (1) Å
*c* = 15.2211 (13) Å
β = 102.246 (7)°
*V* = 3568.8 (5) Å^3^
*Z* = 4
Mo *K*α radiation
μ = 0.21 mm^−1^
*T* = 173 K
0.17 × 0.12 × 0.08 mm

#### Data collection

Stoe IPDS-II two-circle diffractometer
26210 measured reflections
6696 independent reflections
3143 reflections with *I* > 2σ(*I*)
*R*_int_ = 0.097

#### Refinement

*R*[*F* ^2^ > 2σ(*F* ^2^)] = 0.100
*wR*(*F* ^2^) = 0.313
*S* = 1.02
6696 reflections
520 parameters
H-atom parameters constrained
Δρ_max_ = 0.65 e Å^−3^
Δρ_min_ = −0.43 e Å^−3^

### 

Data collection: *X-AREA* (Stoe & Cie, 2001 ▶); cell refinement: *X-AREA*; data reduction: *X-AREA*; program(s) used to solve structure: *SHELXS97* (Sheldrick, 2008 ▶); program(s) used to refine structure: *SHELXL97* (Sheldrick, 2008 ▶); molecular graphics: *XP* (Sheldrick, 2008 ▶); software used to prepare material for publication: *SHELXL97*.

## Supplementary Material

Crystal structure: contains datablocks I, global. DOI: 10.1107/S1600536810044685/hb5716sup1.cif

Structure factors: contains datablocks I. DOI: 10.1107/S1600536810044685/hb5716Isup2.hkl

Additional supplementary materials:  crystallographic information; 3D view; checkCIF report

## Figures and Tables

**Table 1:** Selected bond lengths (Å)

Li1—O3	1.905 (10)
Li1—O1*B*	1.918 (9)
Li1—O1	2.009 (10)
Li1—O1*A*	2.066 (10)
Li2—O2	1.874 (9)
Li2—O3	1.904 (9)
Li2—O1	2.027 (10)
Li2—F53	2.606 (10)
Li2—F83	2.639 (10)
Li3—O2	1.885 (12)
Li3—O1*C*	1.928 (11)
Li3—O1	1.977 (10)
Li3—O1*A*	2.063 (11)

**Fig. 1.**
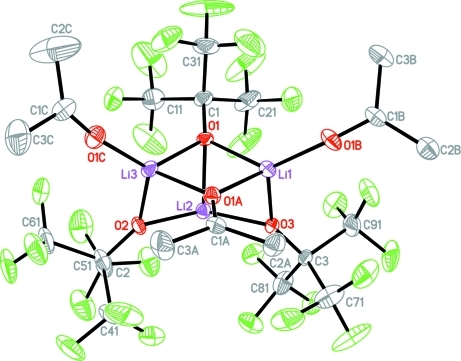
Perspective view of the title compound with displacement ellipsoids drawn at the 20% probability level; H atoms are omitted for clarity.

## supplementary crystallographic information

### Comment

Weakly coordinating anions have gained prominence in wide areas of application in research laboratories and industry. One approach is to create such anions by tetrasubstitution of triele derivatives with perfluorinated alkoxides as [OC(CF_3_)_3_]^-^ (Reisinger *et al.*, 2007) or with siloxides [OSi*R*_3_]^-^ (*R* = alkyl) (Kern *et al.*, 2008; Lerner *et al.*, 2005, 2002). In this paper we report the crystal structure of the adduct of Li[OC(CF_3_)_3_] with acetone, [Li(OC(CH_3_)_2_]_3_[OC(CF_3_)_3_]_3_, (I). According to a literature procedure (Reisinger *et al.*, 2007), Li[OC(CF_3_)_3_] was easily accessible from the reaction of HOC(CF_3_)_3_ with LiH. Single crystals of (I) were obtained by recrystallization from acetone. Only one comparabel cage structure was found in the Cambridge Crystallographic Database, *i.e.* (µ_3_-1,1-diphenylethoxo)-tris(µ_2_-1,1-diphenylethoxo)-bis(1,1-diphenylethoxy)-tri-magnesium toluene solvate (Zechmann *et al.*, 2001). The title compound features an open Li—O cube with an Li ion missing at one corner. Three of the four bridging O atoms carry a *tert*.-butyl residue whereas the fourth is part of an acetone molecule. The two four-coordinated Li atoms are further bonded to an acetone molecule. This is the first example of such an Li—O sceleton and only one comparable structure was determined up to now containing Mg instead of Li as metal atoms.

### Experimental

LiH (0.34 g, 43.7 mmol) and Et_2_O (100 ml) were combined under an N_2_ atmosphere. Li[OC(CF_3_)_3_] (6.1 ml, 43.8 mmol) was added dropwise at ambient temperature. After 1 h at ambient temperature, the solution was heated under reflux for 2 h.  Colourless blocks of (I) were obtained by recrystallization from acetone Yield: 6.1 g (50%).

### Refinement

H atoms were located in a difference map, but geometrically positioned and refined using a riding model with fixed individual displacement parameters [U(H) = 1.2 *U*_eq_(C,*N*)] and with N—H = 0.88Å and C—H = 0.95 Å. The highest peak (2.75 e Å^-3^) in the final difference electron density map is at 1.36Å from Br1.

### Crystal data

**Table:**

[Li_3_(C_4_F_9_O)_3_(C_3_H_6_O)_3_]	*F*(000) = 1776
*M**_r_* = 900.17	*D*_x_ = 1.675 Mg m^−^^3^
Monoclinic, *P*2_1_/*c*	Mo *K*α radiation, λ = 0.71073 Å
Hall symbol: -P 2ybc	Cell parameters from 9264 reflections
*a* = 20.6939 (17) Å	θ = 3.5–25.0°
*b* = 11.594 (1) Å	µ = 0.21 mm^−^^1^
*c* = 15.2211 (13) Å	*T* = 173 K
β = 102.246 (7)°	Block, colourless
*V* = 3568.8 (5) Å^3^	0.17 × 0.12 × 0.08 mm
*Z* = 4	

### Data collection

**Table:**

Stoe IPDS-II two-circle diffractometer	3143 reflections with *I* > 2σ(*I*)
Radiation source: fine-focus sealed tube	*R*_int_ = 0.097
graphite	θ_max_ = 25.7°, θ_min_ = 3.5°
ω scans	*h* = −24→25
26210 measured reflections	*k* = −14→14
6696 independent reflections	*l* = −18→18

### Refinement

**Table:**

Refinement on *F*^2^	Primary atom site location: structure-invariant direct methods
Least-squares matrix: full	Secondary atom site location: difference Fourier map
*R*[*F*^2^ > 2σ(*F*^2^)] = 0.100	Hydrogen site location: inferred from neighbouring sites
*wR*(*F*^2^) = 0.313	H-atom parameters constrained
*S* = 1.02	*w* = 1/[σ^2^(*F*_o_^2^) + (0.1624*P*)^2^ + 1.3913*P*] where *P* = (*F*_o_^2^ + 2*F*_c_^2^)/3
6696 reflections	(Δ/σ)_max_ < 0.001
520 parameters	Δρ_max_ = 0.65 e Å^−^^3^
0 restraints	Δρ_min_ = −0.43 e Å^−^^3^

### Special details

**Table:**

Geometry. All e.s.d.'s (except the e.s.d. in the dihedral angle between two l.s. planes) are estimated using the full covariance matrix. The cell e.s.d.'s are taken into account individually in the estimation of e.s.d.'s in distances, angles and torsion angles; correlations between e.s.d.'s in cell parameters are only used when they are defined by crystal symmetry. An approximate (isotropic) treatment of cell e.s.d.'s is used for estimating e.s.d.'s involving l.s. planes.
Refinement. Refinement of *F*^2^ against ALL reflections. The weighted *R*-factor *wR* and goodness of fit *S* are based on *F*^2^, conventional *R*-factors *R* are based on *F*, with *F* set to zero for negative *F*^2^. The threshold expression of *F*^2^ > σ(*F*^2^) is used only for calculating *R*-factors(gt) *etc*. and is not relevant to the choice of reflections for refinement. *R*-factors based on *F*^2^ are statistically about twice as large as those based on *F*, and *R*- factors based on ALL data will be even larger.

### Fractional atomic coordinates and isotropic or equivalent isotropic displacement parameters (Å^2^)

**Table:**

	*x*	*y*	*z*	*U*_iso_*/*U*_eq_	
Li1	0.6834 (4)	0.5080 (8)	0.4511 (6)	0.043 (2)	
Li2	0.7494 (4)	0.4245 (8)	0.6049 (6)	0.041 (2)	
Li3	0.8063 (5)	0.6077 (8)	0.5565 (7)	0.049 (2)	
O1	0.71434 (17)	0.5853 (3)	0.5708 (3)	0.0415 (9)	
O2	0.83726 (17)	0.4753 (4)	0.6246 (3)	0.0462 (10)	
O3	0.69334 (17)	0.3574 (3)	0.5022 (2)	0.0397 (9)	
C1	0.6790 (3)	0.6422 (6)	0.6218 (5)	0.0565 (17)	
C2	0.8939 (3)	0.4303 (7)	0.6738 (5)	0.0590 (18)	
C3	0.6702 (3)	0.2515 (5)	0.4868 (4)	0.0413 (13)	
C11	0.7219 (5)	0.6635 (11)	0.7182 (7)	0.103 (4)	
C21	0.6158 (5)	0.5731 (9)	0.6284 (9)	0.102 (4)	
C31	0.6578 (5)	0.7626 (9)	0.5792 (8)	0.093 (3)	
C41	0.9234 (4)	0.3436 (9)	0.6161 (9)	0.096 (3)	
C51	0.8799 (4)	0.3704 (9)	0.7572 (6)	0.087 (3)	
C61	0.9451 (4)	0.5287 (10)	0.7049 (7)	0.090 (3)	
C71	0.6859 (6)	0.1984 (8)	0.4035 (7)	0.100 (3)	
C81	0.6918 (5)	0.1737 (7)	0.5691 (5)	0.080 (3)	
C91	0.5902 (4)	0.2547 (8)	0.4709 (7)	0.081 (2)	
O1A	0.77526 (18)	0.5481 (3)	0.4267 (3)	0.0426 (9)	
C1A	0.8023 (3)	0.5270 (5)	0.3646 (4)	0.0457 (14)	
C2A	0.7665 (4)	0.4695 (7)	0.2816 (4)	0.0616 (18)	
H2A1	0.7799	0.3884	0.2823	0.092*	
H2A2	0.7771	0.5079	0.2290	0.092*	
H2A3	0.7188	0.4742	0.2784	0.092*	
C3A	0.8728 (4)	0.5568 (10)	0.3705 (6)	0.091 (3)	
H3A1	0.8897	0.5988	0.4266	0.136*	
H3A2	0.8767	0.6055	0.3192	0.136*	
H3A3	0.8985	0.4860	0.3696	0.136*	
O1B	0.6189 (2)	0.5421 (4)	0.3439 (3)	0.0604 (12)	
C1B	0.5674 (3)	0.5583 (6)	0.2902 (4)	0.0493 (15)	
C2B	0.5452 (4)	0.4790 (7)	0.2126 (5)	0.0660 (19)	
H2B1	0.5761	0.4141	0.2170	0.099*	
H2B2	0.5442	0.5208	0.1564	0.099*	
H2B3	0.5009	0.4500	0.2134	0.099*	
C3B	0.5244 (4)	0.6581 (8)	0.2999 (5)	0.073 (2)	
H3B1	0.4901	0.6335	0.3313	0.110*	
H3B2	0.5037	0.6875	0.2402	0.110*	
H3B3	0.5512	0.7190	0.3345	0.110*	
O1C	0.8575 (2)	0.7423 (4)	0.5414 (4)	0.0727 (14)	
C1C	0.8808 (4)	0.8329 (7)	0.5301 (5)	0.0657 (19)	
C2C	0.8563 (11)	0.9436 (12)	0.5431 (17)	0.227 (11)	
H2C1	0.8137	0.9550	0.5014	0.340*	
H2C2	0.8878	1.0020	0.5318	0.340*	
H2C3	0.8504	0.9506	0.6051	0.340*	
C3C	0.9489 (5)	0.8424 (11)	0.5096 (8)	0.117 (4)	
H3C1	0.9686	0.7654	0.5107	0.176*	
H3C2	0.9768	0.8912	0.5550	0.176*	
H3C3	0.9454	0.8768	0.4500	0.176*	
F11	0.7411 (4)	0.5655 (9)	0.7591 (5)	0.155 (3)	
F12	0.7768 (3)	0.7193 (6)	0.7103 (5)	0.133 (3)	
F13	0.6917 (5)	0.7303 (13)	0.7659 (6)	0.256 (7)	
F21	0.5782 (2)	0.5577 (6)	0.5466 (6)	0.140 (3)	
F22	0.5812 (5)	0.6268 (7)	0.6783 (9)	0.242 (7)	
F23	0.6347 (5)	0.4709 (6)	0.6642 (5)	0.149 (3)	
F31	0.6204 (6)	0.8171 (7)	0.6263 (8)	0.233 (6)	
F32	0.7082 (4)	0.8271 (5)	0.5731 (5)	0.128 (2)	
F33	0.6231 (4)	0.7495 (7)	0.4977 (6)	0.149 (3)	
F41	0.9860 (3)	0.3125 (6)	0.6548 (6)	0.138 (3)	
F42	0.9217 (4)	0.3780 (7)	0.5360 (5)	0.139 (3)	
F43	0.8883 (3)	0.2450 (6)	0.6095 (6)	0.141 (3)	
F51	0.9265 (3)	0.2930 (6)	0.7959 (4)	0.131 (2)	
F52	0.8751 (3)	0.4476 (7)	0.8223 (4)	0.135 (3)	
F53	0.8223 (3)	0.3172 (6)	0.7409 (4)	0.1077 (19)	
F61	0.9188 (3)	0.6205 (6)	0.7306 (5)	0.117 (2)	
F62	0.9717 (2)	0.5628 (6)	0.6347 (5)	0.118 (2)	
F63	0.9970 (2)	0.4962 (6)	0.7679 (4)	0.131 (3)	
F71	0.7528 (3)	0.2176 (5)	0.4061 (4)	0.116 (2)	
F72	0.6800 (5)	0.0836 (5)	0.3979 (5)	0.152 (3)	
F73	0.6503 (3)	0.2477 (5)	0.3268 (3)	0.1021 (18)	
F81	0.7605 (3)	0.1430 (5)	0.5695 (4)	0.1074 (19)	
F82	0.6600 (3)	0.0768 (4)	0.5725 (3)	0.0832 (14)	
F83	0.6956 (2)	0.2306 (4)	0.6451 (2)	0.0793 (13)	
F91	0.5672 (2)	0.3351 (5)	0.4146 (4)	0.0982 (18)	
F92	0.5740 (3)	0.2663 (5)	0.5522 (4)	0.0981 (16)	
F93	0.5642 (3)	0.1539 (5)	0.4341 (4)	0.121 (2)	

### Atomic displacement parameters (Å^2^)

**Table:**

	*U*^11^	*U*^22^	*U*^33^	*U*^12^	*U*^13^	*U*^23^
Li1	0.038 (5)	0.045 (5)	0.045 (5)	0.002 (4)	0.008 (4)	0.005 (4)
Li2	0.037 (4)	0.044 (5)	0.041 (5)	−0.004 (4)	0.009 (4)	−0.003 (4)
Li3	0.039 (5)	0.041 (5)	0.071 (6)	−0.009 (4)	0.023 (4)	−0.016 (5)
O1	0.0337 (18)	0.036 (2)	0.059 (2)	0.0016 (16)	0.0205 (17)	−0.0120 (17)
O2	0.0281 (18)	0.058 (3)	0.049 (2)	0.0018 (18)	−0.0005 (16)	−0.0015 (19)
O3	0.0357 (18)	0.031 (2)	0.050 (2)	−0.0061 (16)	0.0046 (16)	−0.0028 (16)
C1	0.048 (3)	0.047 (4)	0.083 (5)	−0.001 (3)	0.033 (3)	−0.018 (3)
C2	0.034 (3)	0.075 (5)	0.063 (4)	−0.002 (3)	0.001 (3)	0.011 (3)
C3	0.046 (3)	0.034 (3)	0.041 (3)	−0.004 (2)	0.003 (2)	−0.005 (2)
C11	0.083 (6)	0.133 (9)	0.098 (7)	0.018 (7)	0.032 (5)	−0.068 (7)
C21	0.094 (7)	0.086 (7)	0.154 (10)	−0.017 (5)	0.090 (7)	−0.061 (7)
C31	0.076 (6)	0.077 (6)	0.137 (9)	0.030 (5)	0.047 (6)	−0.022 (6)
C41	0.065 (5)	0.085 (7)	0.139 (9)	0.029 (5)	0.025 (5)	0.016 (6)
C51	0.060 (5)	0.104 (7)	0.084 (6)	−0.012 (5)	−0.013 (4)	0.030 (5)
C61	0.039 (4)	0.098 (7)	0.120 (7)	−0.017 (4)	−0.015 (4)	0.022 (6)
C71	0.153 (10)	0.068 (6)	0.093 (7)	−0.007 (6)	0.059 (7)	−0.008 (5)
C81	0.114 (7)	0.057 (5)	0.063 (5)	−0.021 (5)	0.002 (4)	0.011 (4)
C91	0.055 (4)	0.072 (5)	0.110 (7)	−0.019 (4)	0.001 (4)	−0.004 (5)
O1A	0.0373 (19)	0.042 (2)	0.048 (2)	−0.0021 (17)	0.0095 (17)	0.0005 (18)
C1A	0.042 (3)	0.046 (3)	0.052 (3)	0.007 (3)	0.015 (3)	0.005 (3)
C2A	0.067 (4)	0.070 (5)	0.053 (4)	−0.006 (4)	0.023 (3)	−0.013 (3)
C3A	0.052 (4)	0.138 (9)	0.089 (6)	−0.009 (5)	0.034 (4)	−0.018 (6)
O1B	0.041 (2)	0.067 (3)	0.069 (3)	0.007 (2)	0.003 (2)	0.028 (2)
C1B	0.037 (3)	0.053 (4)	0.061 (4)	0.003 (3)	0.017 (3)	0.027 (3)
C2B	0.058 (4)	0.068 (5)	0.072 (5)	0.001 (4)	0.014 (3)	0.016 (4)
C3B	0.059 (4)	0.096 (6)	0.066 (4)	0.030 (4)	0.015 (3)	0.019 (4)
O1C	0.064 (3)	0.051 (3)	0.102 (4)	−0.018 (2)	0.015 (3)	0.007 (3)
C1C	0.089 (5)	0.053 (4)	0.059 (4)	−0.008 (4)	0.024 (4)	−0.004 (3)
C2C	0.27 (2)	0.089 (10)	0.40 (3)	0.021 (12)	0.23 (2)	0.007 (14)
C3C	0.094 (7)	0.132 (10)	0.125 (9)	−0.039 (7)	0.025 (6)	0.014 (7)
F11	0.136 (6)	0.219 (9)	0.121 (5)	0.050 (6)	0.052 (4)	0.078 (6)
F12	0.098 (4)	0.109 (5)	0.167 (6)	0.001 (4)	−0.024 (4)	−0.076 (4)
F13	0.181 (8)	0.435 (18)	0.142 (6)	0.133 (10)	0.012 (6)	−0.161 (9)
F21	0.051 (3)	0.113 (5)	0.246 (9)	−0.016 (3)	0.006 (4)	−0.059 (5)
F22	0.220 (9)	0.161 (7)	0.438 (16)	−0.106 (7)	0.279 (11)	−0.176 (9)
F23	0.260 (9)	0.075 (4)	0.150 (5)	−0.073 (5)	0.132 (6)	−0.033 (4)
F31	0.352 (14)	0.116 (6)	0.310 (12)	0.140 (8)	0.244 (12)	0.044 (7)
F32	0.170 (6)	0.059 (3)	0.166 (6)	−0.030 (4)	0.060 (5)	−0.004 (3)
F33	0.122 (5)	0.146 (6)	0.162 (6)	0.042 (5)	−0.009 (5)	0.050 (5)
F41	0.059 (3)	0.135 (5)	0.215 (7)	0.045 (3)	0.020 (4)	0.038 (5)
F42	0.140 (6)	0.174 (7)	0.121 (5)	0.076 (5)	0.070 (4)	0.008 (5)
F43	0.097 (4)	0.081 (4)	0.234 (8)	0.025 (3)	0.009 (5)	−0.045 (5)
F51	0.094 (4)	0.149 (6)	0.133 (5)	0.008 (4)	−0.013 (3)	0.075 (4)
F52	0.133 (5)	0.202 (8)	0.064 (3)	−0.030 (5)	0.008 (3)	−0.013 (4)
F53	0.081 (3)	0.130 (5)	0.110 (4)	−0.028 (3)	0.014 (3)	0.037 (3)
F61	0.078 (3)	0.093 (4)	0.161 (6)	−0.029 (3)	−0.017 (3)	−0.035 (4)
F62	0.051 (3)	0.131 (5)	0.169 (6)	−0.013 (3)	0.014 (3)	0.049 (4)
F63	0.060 (3)	0.156 (6)	0.146 (5)	−0.031 (3)	−0.051 (3)	0.038 (4)
F71	0.132 (5)	0.099 (4)	0.144 (5)	0.005 (4)	0.093 (4)	−0.020 (4)
F72	0.304 (10)	0.055 (3)	0.136 (5)	−0.038 (4)	0.133 (6)	−0.041 (3)
F73	0.175 (5)	0.089 (3)	0.042 (2)	−0.014 (4)	0.021 (3)	−0.002 (2)
F81	0.088 (3)	0.087 (4)	0.133 (4)	0.037 (3)	−0.007 (3)	0.016 (3)
F82	0.115 (4)	0.045 (2)	0.086 (3)	−0.022 (2)	0.013 (3)	0.019 (2)
F83	0.117 (4)	0.073 (3)	0.043 (2)	0.010 (3)	0.005 (2)	0.004 (2)
F91	0.056 (2)	0.097 (4)	0.123 (4)	−0.017 (2)	−0.023 (3)	0.048 (3)
F92	0.089 (3)	0.119 (4)	0.103 (4)	−0.031 (3)	0.058 (3)	−0.017 (3)
F93	0.117 (4)	0.086 (4)	0.133 (5)	−0.065 (3)	−0.032 (4)	0.009 (3)

### Geometric parameters (Å, °)

**Table:**

Li1—O3	1.905 (10)	C51—F52	1.354 (12)
Li1—O1B	1.918 (9)	C51—F51	1.356 (10)
Li1—O1	2.009 (10)	C61—F61	1.293 (12)
Li1—O1A	2.066 (10)	C61—F63	1.333 (9)
Li1—Li2	2.631 (12)	C61—F62	1.361 (12)
Li1—Li3	2.940 (13)	C71—F72	1.337 (11)
Li2—Li3	2.608 (14)	C71—F73	1.367 (12)
Li2—O2	1.874 (9)	C71—F71	1.395 (13)
Li2—O3	1.904 (9)	C81—F82	1.309 (9)
Li2—O1	2.027 (10)	C81—F83	1.319 (9)
Li2—F53	2.606 (10)	C81—F81	1.464 (11)
Li2—F83	2.639 (10)	C91—F91	1.287 (10)
Li3—O2	1.885 (12)	C91—F92	1.355 (11)
Li3—O1C	1.928 (11)	C91—F93	1.356 (9)
Li3—O1	1.977 (10)	O1A—C1A	1.221 (7)
Li3—O1A	2.063 (11)	C1A—C2A	1.482 (9)
O1—C1	1.347 (7)	C1A—C3A	1.483 (9)
O2—C2	1.355 (7)	C2A—H2A1	0.9800
O3—C3	1.321 (7)	C2A—H2A2	0.9800
C1—C21	1.555 (11)	C2A—H2A3	0.9800
C1—C31	1.563 (13)	C3A—H3A1	0.9800
C1—C11	1.565 (13)	C3A—H3A2	0.9800
C2—C51	1.528 (12)	C3A—H3A3	0.9800
C2—C41	1.542 (13)	O1B—C1B	1.212 (7)
C2—C61	1.560 (11)	C1B—C3B	1.485 (10)
C3—C71	1.505 (11)	C1B—C2B	1.490 (10)
C3—C81	1.531 (9)	C2B—H2B1	0.9800
C3—C91	1.622 (10)	C2B—H2B2	0.9800
C11—F13	1.308 (10)	C2B—H2B3	0.9800
C11—F11	1.315 (14)	C3B—H3B1	0.9800
C11—F12	1.334 (13)	C3B—H3B2	0.9800
C21—F22	1.307 (9)	C3B—H3B3	0.9800
C21—F23	1.329 (14)	O1C—C1C	1.184 (8)
C21—F21	1.332 (13)	C1C—C2C	1.409 (16)
C31—F32	1.302 (11)	C1C—C3C	1.509 (13)
C31—F33	1.303 (12)	C2C—H2C1	0.9800
C31—F31	1.322 (10)	C2C—H2C2	0.9800
C41—F42	1.276 (13)	C2C—H2C3	0.9800
C41—F43	1.346 (12)	C3C—H3C1	0.9800
C41—F41	1.354 (10)	C3C—H3C2	0.9800
C51—F53	1.318 (9)	C3C—H3C3	0.9800
			
O3—Li1—O1B	122.1 (5)	F32—C31—F31	109.5 (10)
O3—Li1—O1	93.0 (4)	F33—C31—F31	107.7 (10)
O1B—Li1—O1	135.2 (6)	F32—C31—C1	112.6 (7)
O3—Li1—O1A	104.8 (4)	F33—C31—C1	109.9 (8)
O1B—Li1—O1A	107.6 (5)	F31—C31—C1	110.3 (9)
O1—Li1—O1A	86.2 (4)	F42—C41—F43	106.8 (11)
O3—Li1—Li2	46.3 (3)	F42—C41—F41	109.2 (9)
O1B—Li1—Li2	165.7 (5)	F43—C41—F41	104.8 (8)
O1—Li1—Li2	49.6 (3)	F42—C41—C2	114.0 (8)
O1A—Li1—Li2	85.3 (4)	F43—C41—C2	108.9 (8)
O3—Li1—Li3	97.6 (4)	F41—C41—C2	112.5 (9)
O1B—Li1—Li3	138.5 (5)	F53—C51—F52	104.1 (9)
O1—Li1—Li3	42.1 (3)	F53—C51—F51	107.6 (8)
O1A—Li1—Li3	44.6 (3)	F52—C51—F51	106.0 (7)
Li2—Li1—Li3	55.5 (3)	F53—C51—C2	112.3 (6)
O2—Li2—O3	131.4 (5)	F52—C51—C2	111.4 (8)
O2—Li2—O1	92.0 (4)	F51—C51—C2	114.8 (8)
O3—Li2—O1	92.5 (4)	F61—C61—F63	110.0 (9)
O2—Li2—F53	69.2 (3)	F61—C61—F62	105.4 (8)
O3—Li2—F53	127.3 (5)	F63—C61—F62	104.3 (7)
O1—Li2—F53	139.3 (5)	F61—C61—C2	113.1 (7)
O2—Li2—Li3	46.3 (3)	F63—C61—C2	113.8 (8)
O3—Li2—Li3	109.5 (5)	F62—C61—C2	109.6 (8)
O1—Li2—Li3	48.5 (3)	F72—C71—F73	109.7 (9)
F53—Li2—Li3	113.2 (4)	F72—C71—F71	103.7 (9)
O2—Li2—Li1	109.3 (5)	F73—C71—F71	108.0 (8)
O3—Li2—Li1	46.3 (3)	F72—C71—C3	115.4 (8)
O1—Li2—Li1	49.0 (3)	F73—C71—C3	112.0 (8)
F53—Li2—Li1	170.6 (5)	F71—C71—C3	107.5 (8)
Li3—Li2—Li1	68.3 (4)	F82—C81—F83	109.3 (7)
O2—Li2—F83	132.4 (5)	F82—C81—F81	106.7 (7)
O3—Li2—F83	68.2 (3)	F83—C81—F81	103.9 (6)
O1—Li2—F83	134.2 (4)	F82—C81—C3	118.4 (6)
F53—Li2—F83	67.1 (3)	F83—C81—C3	112.1 (6)
Li3—Li2—F83	175.8 (5)	F81—C81—C3	105.2 (6)
Li1—Li2—F83	110.8 (4)	F91—C91—F92	114.0 (8)
O2—Li3—O1C	126.4 (5)	F91—C91—F93	107.0 (7)
O2—Li3—O1	93.2 (5)	F92—C91—F93	108.0 (7)
O1C—Li3—O1	133.3 (6)	F91—C91—C3	109.7 (7)
O2—Li3—O1A	104.7 (5)	F92—C91—C3	107.9 (6)
O1C—Li3—O1A	103.0 (5)	F93—C91—C3	110.2 (7)
O1—Li3—O1A	87.1 (4)	C1A—O1A—Li3	135.1 (4)
O2—Li3—Li2	45.9 (3)	C1A—O1A—Li1	133.2 (5)
O1C—Li3—Li2	170.2 (6)	Li3—O1A—Li1	90.8 (4)
O1—Li3—Li2	50.2 (3)	O1A—C1A—C2A	121.5 (5)
O1A—Li3—Li2	86.0 (4)	O1A—C1A—C3A	120.9 (6)
O2—Li3—Li1	97.7 (4)	C2A—C1A—C3A	117.6 (6)
O1C—Li3—Li1	133.4 (6)	C1A—C2A—H2A1	109.5
O1—Li3—Li1	42.9 (3)	C1A—C2A—H2A2	109.5
O1A—Li3—Li1	44.6 (3)	H2A1—C2A—H2A2	109.5
Li2—Li3—Li1	56.2 (3)	C1A—C2A—H2A3	109.5
C1—O1—Li3	130.7 (5)	H2A1—C2A—H2A3	109.5
C1—O1—Li1	129.3 (4)	H2A2—C2A—H2A3	109.5
Li3—O1—Li1	95.0 (4)	C1A—C3A—H3A1	109.5
C1—O1—Li2	120.8 (5)	C1A—C3A—H3A2	109.5
Li3—O1—Li2	81.3 (4)	H3A1—C3A—H3A2	109.5
Li1—O1—Li2	81.4 (4)	C1A—C3A—H3A3	109.5
C2—O2—Li2	131.6 (5)	H3A1—C3A—H3A3	109.5
C2—O2—Li3	140.4 (5)	H3A2—C3A—H3A3	109.5
Li2—O2—Li3	87.9 (4)	C1B—O1B—Li1	163.7 (5)
C3—O3—Li2	131.4 (4)	O1B—C1B—C3B	120.9 (7)
C3—O3—Li1	140.9 (4)	O1B—C1B—C2B	121.2 (6)
Li2—O3—Li1	87.4 (4)	C3B—C1B—C2B	118.0 (6)
O1—C1—C21	110.6 (5)	C1B—C2B—H2B1	109.5
O1—C1—C31	109.7 (6)	C1B—C2B—H2B2	109.5
C21—C1—C31	108.7 (7)	H2B1—C2B—H2B2	109.5
O1—C1—C11	110.3 (6)	C1B—C2B—H2B3	109.5
C21—C1—C11	109.8 (8)	H2B1—C2B—H2B3	109.5
C31—C1—C11	107.6 (8)	H2B2—C2B—H2B3	109.5
O2—C2—C51	109.9 (6)	C1B—C3B—H3B1	109.5
O2—C2—C41	109.7 (6)	C1B—C3B—H3B2	109.5
C51—C2—C41	110.1 (8)	H3B1—C3B—H3B2	109.5
O2—C2—C61	109.8 (6)	C1B—C3B—H3B3	109.5
C51—C2—C61	108.4 (7)	H3B1—C3B—H3B3	109.5
C41—C2—C61	109.0 (7)	H3B2—C3B—H3B3	109.5
O3—C3—C71	113.3 (6)	C1C—O1C—Li3	170.9 (7)
O3—C3—C81	111.7 (5)	O1C—C1C—C2C	128.1 (10)
C71—C3—C81	111.7 (7)	O1C—C1C—C3C	121.5 (8)
O3—C3—C91	109.0 (5)	C2C—C1C—C3C	110.0 (10)
C71—C3—C91	106.3 (7)	C1C—C2C—H2C1	109.5
C81—C3—C91	104.3 (6)	C1C—C2C—H2C2	109.5
F13—C11—F11	112.3 (12)	H2C1—C2C—H2C2	109.5
F13—C11—F12	106.2 (11)	C1C—C2C—H2C3	109.5
F11—C11—F12	106.6 (9)	H2C1—C2C—H2C3	109.5
F13—C11—C1	111.7 (8)	H2C2—C2C—H2C3	109.5
F11—C11—C1	111.2 (9)	C1C—C3C—H3C1	109.5
F12—C11—C1	108.4 (9)	C1C—C3C—H3C2	109.5
F22—C21—F23	109.7 (11)	H3C1—C3C—H3C2	109.5
F22—C21—F21	108.9 (11)	C1C—C3C—H3C3	109.5
F23—C21—F21	109.1 (8)	H3C1—C3C—H3C3	109.5
F22—C21—C1	111.3 (7)	H3C2—C3C—H3C3	109.5
F23—C21—C1	107.8 (9)	C51—F53—Li2	105.9 (5)
F21—C21—C1	110.0 (9)	C81—F83—Li2	99.9 (5)
F32—C31—F33	106.7 (10)		
			
O3—Li1—Li2—O2	129.5 (6)	Li3—O1—C1—C31	74.5 (9)
O1B—Li1—Li2—O2	169 (2)	Li1—O1—C1—C31	−74.1 (8)
O1—Li1—Li2—O2	−75.2 (5)	Li2—O1—C1—C31	−179.3 (6)
O1A—Li1—Li2—O2	13.8 (5)	Li3—O1—C1—C11	−43.9 (10)
Li3—Li1—Li2—O2	−22.0 (4)	Li1—O1—C1—C11	167.5 (7)
O1B—Li1—Li2—O3	39 (2)	Li2—O1—C1—C11	62.3 (8)
O1—Li1—Li2—O3	155.3 (5)	Li2—O2—C2—C51	−33.8 (10)
O1A—Li1—Li2—O3	−115.7 (5)	Li3—O2—C2—C51	140.4 (8)
Li3—Li1—Li2—O3	−151.4 (5)	Li2—O2—C2—C41	87.4 (8)
O3—Li1—Li2—O1	−155.3 (5)	Li3—O2—C2—C41	−98.4 (9)
O1B—Li1—Li2—O1	−116 (2)	Li2—O2—C2—C61	−152.9 (7)
O1A—Li1—Li2—O1	89.0 (4)	Li3—O2—C2—C61	21.3 (11)
Li3—Li1—Li2—O1	53.3 (3)	Li2—O3—C3—C71	−124.9 (7)
O3—Li1—Li2—Li3	151.4 (5)	Li1—O3—C3—C71	64.3 (10)
O1—Li1—Li2—Li3	−53.3 (3)	Li2—O3—C3—C81	2.4 (9)
O1A—Li1—Li2—Li3	35.8 (3)	Li1—O3—C3—C81	−168.5 (7)
O3—Li1—Li2—F83	−24.2 (4)	Li2—O3—C3—C91	117.1 (7)
O1—Li1—Li2—F83	131.1 (5)	Li1—O3—C3—C91	−53.8 (9)
O1A—Li1—Li2—F83	−139.9 (4)	O1—C1—C11—F13	172.4 (11)
Li3—Li1—Li2—F83	−175.6 (5)	C21—C1—C11—F13	−65.4 (14)
O3—Li2—Li3—O2	−129.2 (6)	C31—C1—C11—F13	52.8 (14)
O1—Li2—Li3—O2	155.4 (6)	O1—C1—C11—F11	−61.2 (10)
F53—Li2—Li3—O2	19.2 (4)	C21—C1—C11—F11	61.0 (9)
Li1—Li2—Li3—O2	−150.8 (5)	C31—C1—C11—F11	179.2 (8)
O2—Li2—Li3—O1	−155.4 (6)	O1—C1—C11—F12	55.7 (10)
O3—Li2—Li3—O1	75.4 (5)	C21—C1—C11—F12	177.9 (7)
F53—Li2—Li3—O1	−136.2 (5)	C31—C1—C11—F12	−63.9 (9)
Li1—Li2—Li3—O1	53.9 (3)	O1—C1—C21—F22	179.5 (11)
O2—Li2—Li3—O1A	115.0 (5)	C31—C1—C21—F22	−60.0 (14)
O3—Li2—Li3—O1A	−14.2 (5)	C11—C1—C21—F22	57.5 (14)
O1—Li2—Li3—O1A	−89.6 (4)	O1—C1—C21—F23	59.1 (10)
F53—Li2—Li3—O1A	134.2 (4)	C31—C1—C21—F23	179.6 (7)
Li1—Li2—Li3—O1A	−35.8 (3)	C11—C1—C21—F23	−62.9 (9)
O2—Li2—Li3—Li1	150.8 (5)	O1—C1—C21—F21	−59.7 (10)
O3—Li2—Li3—Li1	21.5 (4)	C31—C1—C21—F21	60.8 (9)
O1—Li2—Li3—Li1	−53.9 (3)	C11—C1—C21—F21	178.3 (7)
F53—Li2—Li3—Li1	169.9 (5)	O1—C1—C31—F32	−60.3 (10)
O3—Li1—Li3—O2	0.3 (5)	C21—C1—C31—F32	178.6 (8)
O1B—Li1—Li3—O2	−163.3 (7)	C11—C1—C31—F32	59.7 (10)
O1—Li1—Li3—O2	86.4 (5)	O1—C1—C31—F33	58.4 (9)
O1A—Li1—Li3—O2	−103.2 (5)	C21—C1—C31—F33	−62.6 (9)
Li2—Li1—Li3—O2	20.7 (4)	C11—C1—C31—F33	178.5 (8)
O3—Li1—Li3—O1C	162.3 (6)	O1—C1—C31—F31	177.0 (9)
O1B—Li1—Li3—O1C	−1.3 (12)	C21—C1—C31—F31	56.0 (12)
O1—Li1—Li3—O1C	−111.6 (8)	C11—C1—C31—F31	−62.9 (11)
O1A—Li1—Li3—O1C	58.8 (7)	O2—C2—C41—F42	43.0 (11)
Li2—Li1—Li3—O1C	−177.3 (8)	C51—C2—C41—F42	164.0 (8)
O3—Li1—Li3—O1	−86.1 (5)	C61—C2—C41—F42	−77.2 (10)
O1B—Li1—Li3—O1	110.3 (9)	O2—C2—C41—F43	−76.2 (9)
O1A—Li1—Li3—O1	170.4 (6)	C51—C2—C41—F43	44.8 (9)
Li2—Li1—Li3—O1	−65.7 (4)	C61—C2—C41—F43	163.6 (8)
O3—Li1—Li3—O1A	103.5 (5)	O2—C2—C41—F41	168.0 (8)
O1B—Li1—Li3—O1A	−60.1 (7)	C51—C2—C41—F41	−71.0 (10)
O1—Li1—Li3—O1A	−170.4 (6)	C61—C2—C41—F41	47.8 (11)
Li2—Li1—Li3—O1A	123.9 (5)	O2—C2—C51—F53	37.0 (11)
O3—Li1—Li3—Li2	−20.4 (4)	C41—C2—C51—F53	−83.9 (10)
O1B—Li1—Li3—Li2	176.0 (9)	C61—C2—C51—F53	157.0 (8)
O1—Li1—Li3—Li2	65.7 (4)	O2—C2—C51—F52	−79.3 (8)
O1A—Li1—Li3—Li2	−123.9 (5)	C41—C2—C51—F52	159.8 (7)
O2—Li3—O1—C1	106.0 (6)	C61—C2—C51—F52	40.7 (9)
O1C—Li3—O1—C1	−44.3 (11)	O2—C2—C51—F51	160.3 (7)
O1A—Li3—O1—C1	−149.4 (6)	C41—C2—C51—F51	39.4 (10)
Li2—Li3—O1—C1	123.4 (7)	C61—C2—C51—F51	−79.7 (10)
Li1—Li3—O1—C1	−156.1 (7)	O2—C2—C61—F61	41.1 (11)
O2—Li3—O1—Li1	−97.9 (4)	C51—C2—C61—F61	−78.9 (10)
O1C—Li3—O1—Li1	111.9 (8)	C41—C2—C61—F61	161.2 (8)
O1A—Li3—O1—Li1	6.7 (4)	O2—C2—C61—F63	167.5 (8)
Li2—Li3—O1—Li1	−80.4 (4)	C51—C2—C61—F63	47.5 (12)
O2—Li3—O1—Li2	−17.4 (4)	C41—C2—C61—F63	−72.4 (11)
O1C—Li3—O1—Li2	−167.7 (8)	O2—C2—C61—F62	−76.2 (9)
O1A—Li3—O1—Li2	87.2 (4)	C51—C2—C61—F62	163.8 (7)
Li1—Li3—O1—Li2	80.4 (4)	C41—C2—C61—F62	44.0 (9)
O3—Li1—O1—C1	−105.4 (6)	O3—C3—C71—F72	162.5 (9)
O1B—Li1—O1—C1	38.6 (11)	C81—C3—C71—F72	35.3 (13)
O1A—Li1—O1—C1	149.9 (5)	C91—C3—C71—F72	−77.9 (11)
Li2—Li1—O1—C1	−123.0 (6)	O3—C3—C71—F73	−71.1 (10)
Li3—Li1—O1—C1	156.6 (7)	C81—C3—C71—F73	161.6 (7)
O3—Li1—O1—Li3	98.0 (5)	C91—C3—C71—F73	48.5 (9)
O1B—Li1—O1—Li3	−118.0 (8)	O3—C3—C71—F71	47.4 (9)
O1A—Li1—O1—Li3	−6.7 (4)	C81—C3—C71—F71	−79.8 (8)
Li2—Li1—O1—Li3	80.3 (4)	C91—C3—C71—F71	167.0 (7)
O3—Li1—O1—Li2	17.6 (4)	O3—C3—C81—F82	163.0 (7)
O1B—Li1—O1—Li2	161.6 (8)	C71—C3—C81—F82	−68.9 (11)
O1A—Li1—O1—Li2	−87.1 (4)	C91—C3—C81—F82	45.4 (10)
Li3—Li1—O1—Li2	−80.3 (4)	O3—C3—C81—F83	34.3 (9)
O2—Li2—O1—C1	−115.0 (5)	C71—C3—C81—F83	162.3 (8)
O3—Li2—O1—C1	113.3 (5)	C91—C3—C81—F83	−83.3 (8)
F53—Li2—O1—C1	−55.4 (8)	O3—C3—C81—F81	−78.0 (7)
Li3—Li2—O1—C1	−132.5 (5)	C71—C3—C81—F81	50.1 (8)
Li1—Li2—O1—C1	130.9 (5)	C91—C3—C81—F81	164.4 (6)
F83—Li2—O1—C1	51.8 (8)	O3—C3—C91—F91	47.9 (8)
O2—Li2—O1—Li3	17.5 (4)	C71—C3—C91—F91	−74.4 (8)
O3—Li2—O1—Li3	−114.1 (5)	C81—C3—C91—F91	167.4 (7)
F53—Li2—O1—Li3	77.2 (7)	O3—C3—C91—F92	−76.8 (7)
Li1—Li2—O1—Li3	−96.5 (4)	C71—C3—C91—F92	160.8 (7)
F83—Li2—O1—Li3	−175.6 (6)	C81—C3—C91—F92	42.6 (8)
O2—Li2—O1—Li1	114.0 (4)	O3—C3—C91—F93	165.4 (6)
O3—Li2—O1—Li1	−17.6 (4)	C71—C3—C91—F93	43.0 (9)
F53—Li2—O1—Li1	173.7 (7)	C81—C3—C91—F93	−75.1 (8)
Li3—Li2—O1—Li1	96.5 (4)	O2—Li3—O1A—C1A	−83.6 (7)
F83—Li2—O1—Li1	−79.1 (6)	O1C—Li3—O1A—C1A	50.0 (8)
O3—Li2—O2—C2	−106.8 (8)	O1—Li3—O1A—C1A	−176.2 (6)
O1—Li2—O2—C2	158.1 (6)	Li2—Li3—O1A—C1A	−125.9 (6)
F53—Li2—O2—C2	15.2 (7)	Li1—Li3—O1A—C1A	−169.7 (7)
Li3—Li2—O2—C2	176.3 (7)	O2—Li3—O1A—Li1	86.0 (5)
Li1—Li2—O2—C2	−154.9 (6)	O1C—Li3—O1A—Li1	−140.4 (5)
F83—Li2—O2—C2	−9.1 (10)	O1—Li3—O1A—Li1	−6.5 (4)
O3—Li2—O2—Li3	76.9 (7)	Li2—Li3—O1A—Li1	43.8 (4)
O1—Li2—O2—Li3	−18.2 (4)	O3—Li1—O1A—C1A	84.3 (7)
F53—Li2—O2—Li3	−161.1 (4)	O1B—Li1—O1A—C1A	−47.1 (8)
Li1—Li2—O2—Li3	28.7 (5)	O1—Li1—O1A—C1A	176.4 (5)
F83—Li2—O2—Li3	174.6 (6)	Li2—Li1—O1A—C1A	126.7 (6)
O1C—Li3—O2—C2	−3.6 (12)	Li3—Li1—O1A—C1A	170.0 (7)
O1—Li3—O2—C2	−157.0 (7)	O3—Li1—O1A—Li3	−85.7 (5)
O1A—Li3—O2—C2	115.1 (8)	O1B—Li1—O1A—Li3	142.9 (5)
Li2—Li3—O2—C2	−175.7 (9)	O1—Li1—O1A—Li3	6.4 (4)
Li1—Li3—O2—C2	160.1 (7)	Li2—Li1—O1A—Li3	−43.3 (4)
O1C—Li3—O2—Li2	172.0 (7)	Li3—O1A—C1A—C2A	171.8 (6)
O1—Li3—O2—Li2	18.7 (4)	Li1—O1A—C1A—C2A	6.1 (10)
O1A—Li3—O2—Li2	−69.2 (5)	Li3—O1A—C1A—C3A	−7.4 (11)
Li1—Li3—O2—Li2	−24.2 (4)	Li1—O1A—C1A—C3A	−173.1 (7)
O2—Li2—O3—C3	109.3 (8)	O3—Li1—O1B—C1B	68 (2)
O1—Li2—O3—C3	−155.8 (5)	O1—Li1—O1B—C1B	−68 (2)
F53—Li2—O3—C3	14.9 (9)	O1A—Li1—O1B—C1B	−171.0 (18)
Li3—Li2—O3—C3	157.6 (5)	Li2—Li1—O1B—C1B	35 (4)
Li1—Li2—O3—C3	−174.2 (7)	Li3—Li1—O1B—C1B	−131.3 (18)
F83—Li2—O3—C3	−18.6 (6)	Li1—O1B—C1B—C3B	67 (2)
O2—Li2—O3—Li1	−76.4 (7)	Li1—O1B—C1B—C2B	−113 (2)
O1—Li2—O3—Li1	18.4 (4)	F52—C51—F53—Li2	94.9 (6)
F53—Li2—O3—Li1	−170.8 (6)	F51—C51—F53—Li2	−153.0 (7)
Li3—Li2—O3—Li1	−28.1 (5)	C2—C51—F53—Li2	−25.7 (10)
F83—Li2—O3—Li1	155.6 (4)	O2—Li2—F53—C51	9.0 (7)
O1B—Li1—O3—C3	3.8 (11)	O3—Li2—F53—C51	135.9 (8)
O1—Li1—O3—C3	154.6 (6)	O1—Li2—F53—C51	−58.3 (10)
O1A—Li1—O3—C3	−118.5 (7)	Li3—Li2—F53—C51	−5.7 (8)
Li2—Li1—O3—C3	173.1 (8)	F83—Li2—F53—C51	169.7 (7)
Li3—Li1—O3—C3	−163.4 (6)	F82—C81—F83—Li2	−174.1 (6)
O1B—Li1—O3—Li2	−169.3 (6)	F81—C81—F83—Li2	72.3 (5)
O1—Li1—O3—Li2	−18.6 (4)	C3—C81—F83—Li2	−40.7 (7)
O1A—Li1—O3—Li2	68.3 (5)	O2—Li2—F83—C81	−94.5 (8)
Li3—Li1—O3—Li2	23.4 (4)	O3—Li2—F83—C81	32.4 (5)
Li3—O1—C1—C21	−165.6 (8)	O1—Li2—F83—C81	103.4 (7)
Li1—O1—C1—C21	45.8 (10)	F53—Li2—F83—C81	−119.1 (5)
Li2—O1—C1—C21	−59.4 (9)	Li1—Li2—F83—C81	51.0 (6)
